# Association analysis between variants in *KISS1* gene and litter size in goats

**DOI:** 10.1186/1471-2156-14-63

**Published:** 2013-08-02

**Authors:** Xiaopeng An, Teng Ma, Jinxing Hou, Fang Fang, Peng Han, Yan Yan, Haibo Zhao, Yunxuan Song, Jiangang Wang, Binyun Cao

**Affiliations:** 1 College of Animal Science and Technology, Northwest A&F University, Yangling, Shaanxi 712100, P.R. China

**Keywords:** Combinative genotype, SNP, PCR-RFLP, Candidate gene

## Abstract

**Background:**

Kisspeptins are the peptide products of *KISS1* gene, which operate via the G - protein-coupled receptor GPR54. These peptides have emerged as essential upstream regulators of neurons secreting gonadotropin-releasing hormone (GnRH), the major hypothalamic node for the stimulatory control of the hypothalamic–pituitary– gonadal (HPG) axis. The present study detected the polymorphisms of caprine *KISS1* gene in three goat breeds and investigated the associations between these genetic markers and litter size.

**Results:**

Three goat breeds (n = 680) were used to detect single nucleotide polymorphisms (SNPs) in the coding regions with their intron–exon boundaries and the proximal flanking regions of *KISS1* gene by DNA sequencing and PCR–RFLP. Eleven novel SNPs (g.384G>A, g.1147T>C, g.1417G>A, g.1428_1429delG, g.2124C>T, g.2270C>T, g.2489T>C, g.2510G>A, g.2540C>T, g.3864_3865delCA and g.3885_3886insACCCC) were identified. It was shown that Xinong Saanen and Guanzhong goat breeds were in Hardy-Weinberg disequilibrium at *g.384G>A* locus (*P* < 0.05). Both *g.2510G>A* and *g.2540C>T* loci were closely linked in Xinong Saanen (SN), Guanzhong (GZ) and Boer (BG) goat breeds (*r*^*2*^ > 0.33). The g.384G>A, g.2489T>C, g.2510G>A and g.2540C>T SNPs were associated with litter size (*P*<0.05). Individuals with *AATTAATT* combinative genotype of SN breed (SC) and *TTAATT* combinative genotype of BG breed (BC) had higher litter size than those with other combinative genotypes in average parity. The results extend the spectrum of genetic variation of the caprine *KISS1* gene, which might contribute to goat genetic resources and breeding.

**Conclusions:**

This study explored the genetic polymorphism of *KISS1* gene, and indicated that four SNPs may play an important role in litter size. Their genetic mechanism of reproduction in goat breeds should be further investigated. The female goats with SC1 (*AATTAATT*) and BC7 (*TTAATT*) had higher litter size than those with other combinative genotypes in average parity and could be used for the development of new breeds of prolific goats. Further research on a large number of animals is required to confirm the link with increased prolificacy in goats.

## Background

Kisspeptins are the peptide products of *KISS1* gene, which operate via the G - protein-coupled receptor GPR54 (also known as KISS1R). These peptides have emerged as essential upstream regulators of neurons secreting gonadotropin-releasing hormone (GnRH), the major hypothalamic node for the stimulatory control of the hypothalamic–pituitary– gonadal (HPG) axis [1]. They are potent elicitors of gonadotropin secretion in various species and physiological settings. Moreover, KISS1 neurons in the hypothalamus participate in crucial features of reproductive maturation and function, such as brain-level sex differentiation, puberty onset and the neuroendocrine regulation of gonadotropin secretion and ovulation [2]. Irwig et al. (2004) and Navarro et al. (2004) have provided evidences in rats that kisspeptin-expressing neurons are targets for regulation by sex steroids [3,4], furthermore, these neurons are directly regulated by the negative and positive feedback actions of sex steroids in distinct regions of the forebrain [5].

Mutations of *KISS1R* are associated with hypogonadotrophic hypogonadism in humans [6,7], a phenotype which is also observed in mice carrying inactivating mutations of *KISS1* or *KISS1R* genes [8]. In addition, to their prominent expression at hypothalamic levels, fragmentary evidences suggest that KISS1 and/or KISS1R mRNAs or proteins are also present in several peripheral reproductive tissues including the ovary [9,10], oviduct [11] and testes [12]. In humans, Pinto et al. (2012) reported kisspeptin modulated sperm progressive motility causing a biphasic (stimulatory and inhibitory) response and also induced transient sperm hyperactivation [13]. One novel nonsynonymous single nucleotide polymorphism (G54650055T) substituting one amino acid in kisspeptin (P110T) was found to be statistically related to central precocious puberty (*P*<0.025) in humans [14]. In sheep, KISS1 mRNA-expressing cells are found in the arcuate nucleus (ARC) and dorsallateral preoptic area and both appear to mediate the positive feedback effect of estradiol to generate the preovulatory GnRH/LH surge [15]. The LH surge has been associated with an increase in the LH response to kisspeptin in humans and sheep [16,17], indicating the surge may be generated by increased kisspeptin output and sensitivity. These findings indicate that *KISS1* gene is an excellent candidate gene for reproductive traits in human and livestock.

Based on above considerations, here we detected the polymorphisms of caprine *KISS1* gene in three goat breeds and investigated the associations between these genetic markers and litter size. This study provides some useful information on goat genetic resources and breeding.

## Results

### SNPs identification and genotypes

**Figure 1:**
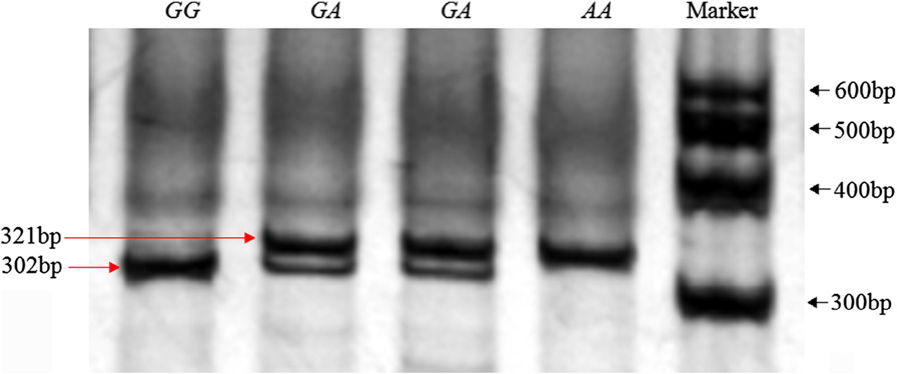
**The electrophoresis patterns obtained after digestion with *MwoI *endonuclease at *g.*384*G>A *locus.***Note*: Fragments including 19 bp of *GG* and *GA* genotypes were invisible.

**Figure 2:**
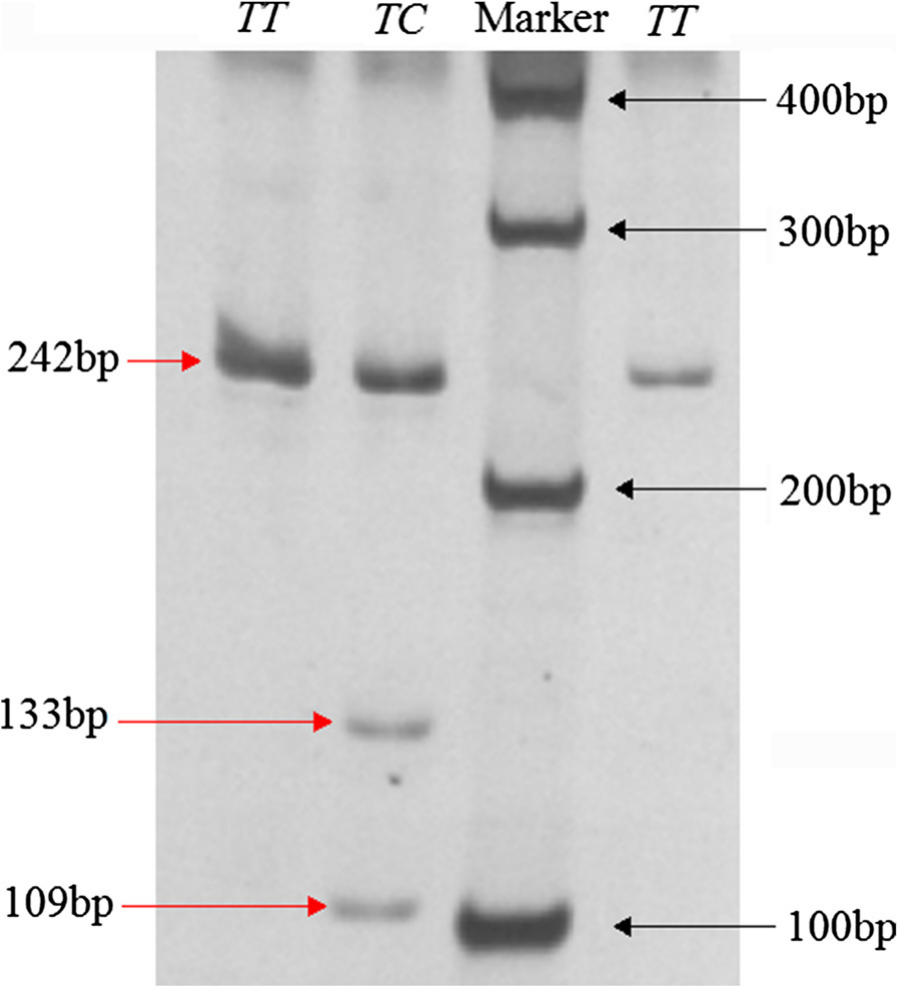
**The electrophoresis patterns obtained after digestion with *SfaN*I endonuclease at g.2489*T*>*C *locus.**

**Figure 3:**
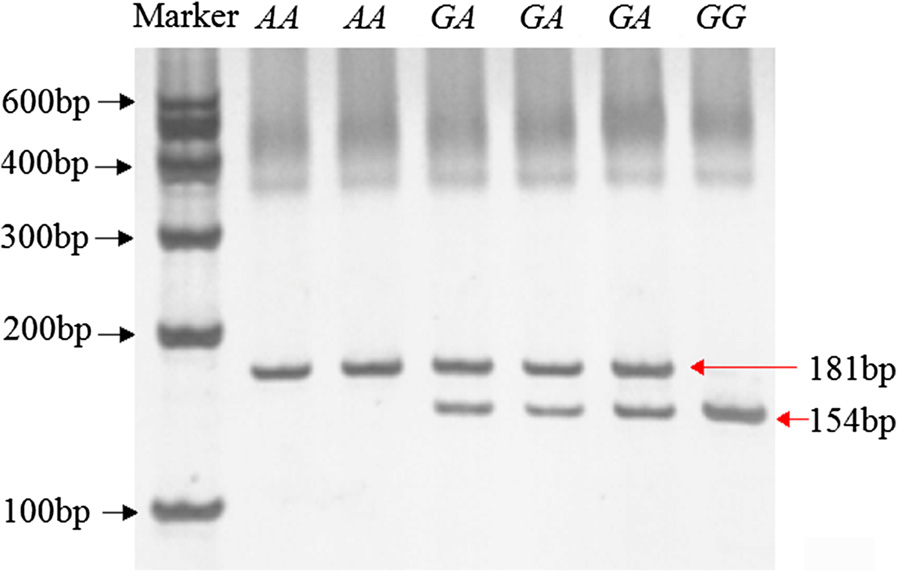
**The electrophoresis patterns obtained after digestion with *Alu*I endonuclease at g.2510*G*>*A *locus.***Note*: Fragments including 61 bp of three genotypes and 27bp of *GG* an *GA* genotypes were invisible.

**Figure 4:**
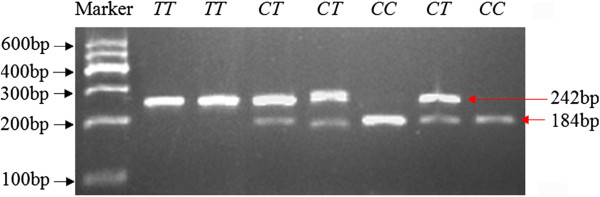
**The electrophoresis patterns obtained after digestion with *Sac*I endonuclease at g.2540*C*>*T *locus.***Note*: Fragments including 58 bp of CC and CT genotypes were invisible.

In the current study, sequencing of the amplicons of different primer pairs identified eleven polymorphic nucleotide sites in caprine *KISS1* gene. The g.384G>A mutation was in the 5′UTR (Additional file 1: Table S1), which was not found in BG breed. The g.3864_3865delCA and g.3885_3886insACCCC mutations were in the 3′UTR. Other mutations were in the intron 1 (g.1147T>C, g.1417G>A, g.1428_1429delG, g.2124C>T, g.2270C>T, g.2489T>C, g.2510G>A and g.2540C>T). SNP accession number is showed in Additional file 1: Table S1. Four SNPs (g.384G>A, g.2489T>C, g.2510G>A and g.2540C>T) were genotyped in three goat breeds (Figures 1, 2, 3, and 4). At *g.384G>A* locus, the PIC was 0.37 in SN and GZ breeds (Additional file 1: Table S2). At *g.2489T>C* locus, the PIC was 0.24–0.29 in three goat breeds. At other two loci, the PIC was 0.36–0.38 in three goat breeds. Genotypic distribution and allelic frequencies of four SNPs are shown in Additional file 1: Table S2. It was shown that SN and GZ breeds were in Hardy-Weinberg disequilibrium at *g.384G>A* locus (*P* < 0.05) (Additional file 1: Table S2). To reveal the linkage relationships between the four SNPs, the linkage disequilibrium was estimated in these breeds (Additional file 1: Table S3). If *r*^*2*^ > 0.33, the linkage disequilibrium was considered strong [18]. Following the result, both *g.2510G>A* and *g.2540C>T* loci were closely linked in three goat breeds (Additional file 1: Table S3).

### Association analysis of SNPs with litter size

At *g.384G>A* locus in SN breed (Table 1), individuals with *AA* genotype had greater litter size than those with *GG* genotype in the second, third and average parity (*P* < 0.05); in GZ breed, individuals with *AA* genotype had greater litter size than those with *GG* genotype in the third and average parity (*P* < 0.05), and individuals with *GA* genotype had greater litter size than those with *GG* genotype in the first and average parity (*P* < 0.05). At *g.2510G>A* locus in SN breed (Table 2), individuals with *AA* genotype had greater litter size than those with *GA* genotype in the third parity (*P* < 0.05); in BG breed, individuals with *AA* genotype had greater litter size than those with *GG* genotype in the fourth and average parity (*P* < 0.05). At *g.2540C>T* locus in GZ breed (Table 2), individuals with *TT* genotype had greater litter size than those with *CC* genotype in the fourth parity (*P* < 0.05); in BG breed, individuals with *TT* genotype had greater litter size than those with *CC* and *CT* genotypes in average parity (*P* < 0.05).

**Table 1:** **Least square means and standard errors of the litter size of SN and GZ breeds at *g*.384 *G *>*A *locus**

**Locus**	**Breed**	**Genotype**	**Number**	**1**^**st **^**parity litter size**	**2**^**nd **^**parity litter size**	**3**^**rd **^**parity litter size**	**4**^**th**^**parity litter size**	**Average litter size**
*g.384G>A*	SN	*AA*	44	1.57±0.09	2.02±0.08^b^	2.22±0.09^b^	2.21±0.10	2.00±0.05^b^
		*GA*	144	1.44±0.05	1.97±0.05^b^	2.03±0.06	2.19±0.07	1.91±0.03
		*GG*	69	1.48±0.07	1.79±0.07^a^	1.98±0.07^a^	2.21±0.08	1.86±0.04^a^
	GZ	*AA*	34	1.46±0.09	1.62±0.09	2.00±0.09^b^	1.94±0.09	1.75±0.04^b^
		*GA*	130	1.48±0.05^b^	1.65±0.04	1.77±0.05^a^	1.94±0.05	1.71±0.02^b^
		*GG*	67	1.33±0.06^a^	1.61±0.06	1.70±0.07^a^	1.91±0.07	1.63±0.03^a^

*Note*: Values with different superscripts within the same column in particular breed differ significantly at *P* < 0.05.

**Table 2:** **Least square means and standard errors of the litter size of three breeds at *g*.2489 *T *>*C *, *g*.2510 *G *>*A *and *g*.2540 *C *>*T *loci**

**Locus**	**Breed**	**Genotype**	**Number**	**1**^**st **^**parity litter size**	**2**^**nd **^**parity litter size**	**3**^**rd **^**parity litter size**	**4**^**th**^**parity litter size**	**Average litter size**
*g.2489T>C*	SN	*TC*	92	1.51±0.06	1.95±0.06	2.04±0.07	2.21±0.07	1.94±0.04
		*TT*	165	1.45±0.05	1.92±0.05	2.05±0.05	2.19±0.06	1.90±0.03
	GZ	*TC*	105	1.36±0.05^a^	1.68±0.05	1.80±0.06	1.89±0.06	1.69±0.03
		*TT*	126	1.50±0.05^b^	1.60±0.05	1.78±0.06	1.97±0.05	1.70±0.02
	BG	*TC*	65	1.32±0.06	1.78±0.07	1.84±0.07	1.98±0.07	1.72±0.04
		*TT*	127	1.29±0.05	1.73±0.05	1.87±0.06	2.09±0.06	1.72±0.03
*g.2510G>A*	SN	*AA*	95	1.49±0.06	1.91±0.06	2.12±0.07^b^	2.27±0.08	1.94±0.04
		*GA*	130	1.47±0.05	1.95±0.05	1.96±0.06^a^	2.15±0.07	1.88±0.31
		*GG*	32	1.44±0.10	1.93±0.09	2.1±0.11	2.17±0.12	1.95±0.06
	GZ	*AA*	55	1.52±0.07^b^	1.54±0.07	1.69±0.08	1.97±0.07	1.68±0.04
		*GA*	118	1.36±0.05^a^	1.67±0.05	1.84±0.06	1.94±0.05	1.70±0.02
		*GG*	58	1.51±0.07	1.65±0.05	1.79±0.07	1.88±0.07	1.71±0.03
	BG	*AA*	71	1.37±0.06	1.79±0.06	2.01±0.07^b^	2.20±0.07^b^	1.82±0.03^b^
		*GA*	89	1.24±0.05	1.73±0.06	1.75±0.06^a^	1.97±0.06^a^	1.65±0.03^a^
		*GG*	32	1.35±0.09	1.73±0.09	1.87±0.10	1.87±0.09^a^	1.69±0.05^a^
*g.2540C>T*	SN	*CC*	42	1.43±0.09	1.87±0.08	2.00±0.10	2.18±0.11	1.90±0.05
		*CT*	121	1.52±0.05	1.96±0.05	1.99±0.06	2.17±0.07	1.93±0.03
		*TT*	94	1.42±0.06	1.94±0.06	2.00±0.07	2.24±0.08	1.90±0.04
	*GZ*	*CC*	47	1.52±0.08	1.68±0.07	1.69±0.08	1.78±0.08^a^	1.67±0.04
		*CT*	108	1.38±0.05	1.62±0.05	1.86±0.06	1.94±0.05	1.69±0.03
		*TT*	76	1.46±0.06	1.63±0.06	1.77±0.07	2.00±0.06^b^	1.71±0.03
	BG	*CC*	40	1.30±0.08	1.71±0.09	1.85±0.09	2.01±0.09	1.69±0.05^a^
		*CT*	89	1.25±0.05	1.76±0.06	1.78±0.06^a^	1.98±0.06^a^	1.67±0.03^a^
		*TT*	63	1.38±0.06	1.75±0.07	2.00±0.07^b^	2.19±0.08^b^	1.81±0.04^b^

*Note*: Values with different superscripts within the same column and mutation locus in particular breed differ significantly at *P* < 0.05.

### Effects of combinative genotypes on litter size

Association analysis of combinative genotypes of *g.384G>A*, *g.2489T>C*, *g.2510G>A* and *g.2540C>T* loci with litter size was done in SN and GZ breeds. In SN breed (Additional file 1: Table S4), individuals with SC3 (*AATCGACT*) had the greatest litter size in comparison with other combinative genotypes in the first parity. Individuals with SC1 (*AATTAATT*) and SC3 (*AATCGACT*) had greater litter size than those with SC4 (*AATTGACT*) and SC10 (*GGTTAATT*) in the second parity (*P* < 0.05). Individuals with SC1 (*AATTAATT*) had greater litter size than those with SC2 (*AATTGGCC*) and SC4 (*AATTGACT*) in the fourth parity (*P* < 0.05). Individuals with SC3 (*AATCGACT*) had greater litter size than those with SC4 (*AATTGACT*), SC5 (*GATCGGCC*) and SC13 (*GATTGGCC*) in average parity (*P* < 0.05). In GZ breed (Additional file 1: Table S5), Individuals with GC2 (*GATCGGCC*) had greater litter size than those with GC12 (*GGTCGACT*) and GC14 (*GGTCGATT*) in the first parity (*P* < 0.05). Individuals with GC14 (*GGTCGATT*) had the lowest litter size in comparison with other combinative genotypes in the third parity. Association analysis of combinative genotypes of *g.2489T>C*, *g.2510G>A* and *g.2540C>T* loci with litter size was done in BG breeds. In BG breed (Additional file 1: Table S6), individuals with BC7(*TTAATT*) had greater litter size than those with BC6(*TTGACT*) in the third, fourth and average parity (*P* < 0.05). Individuals with BC6 (*TTGACT*) had the lowest litter size in comparison with other combinative genotypes in average parity.

## Discussion

According to the classification of PIC (low polymorphism if PIC value < 0.25, moderate polymorphism if 0.25 < PIC value < 0.50, and high polymorphism if PIC > 0.50), SN and GZ breeds at *g.384G>A* locus had moderate genetic diversity, and SN, GZ and BG breeds had also moderate genetic diversity at *g.2510G>A* and *g.2540C>T* loci. The *g.384G>A* locus was in Hardy–Weinberg disequilibrium in SN and GZ breeds (*P*<0.05), which showed the genotypic frequencies were affected by selection, mutation or migration.

Identification of the candidate genes that are responsible for variation in continuous traits or quantitative traits has been a challenge in modern genetics. So far, there have been some studies of *KISS1* gene as a candidate gene on reproductive traits in animals, which revealed that *KISS1* gene plays an important role in animal reproduction [18-21]. Both c.374C>T and c.422C>G mutations were identified in human *KISS1* gene, and the c.374C>T variant was associated with higher kisspeptin resistance to degradation in comparison with the wild type, suggesting a role for this mutation in the precocious puberty phenotype [22]. Huijbregts et al. (2012) detected three SNPs (c.638insT, c.641C>G and c.645G>CA) in the 3′UTR of human *KISS1* gene, and the c.645G>CA mutation was associated with central precocious puberty [20]. In Jining Grey goat *KISS1* gene, there are two mutations (G3433A and C3688A) in exon 3, three mutations (G296C, G454T and T505A) in intron 1 and an 18 bp deletion/insertion (1960–1977) in intron 2 and no mutations in exon 2 [23]. Feng et al. (2009) detected the polymorphism in exon 2 of goat *KISS1* gene and did not find polymorphism [24]. In the current study, ten polymorphisms were detected in *KISS1* gene of three goat breeds (g.1147T>C, g.1417G>A, g.1428_1429delG, g.2124C>T, g.2270C>T, g.2489T>C, g.2510G>A, g.2540C>T, g.3864_3865delCA and g.3885_3886insACCCC). The g.384G>A mutation was detected in SN and GZ breeds. Cao et al. (2010) indicated an association between allele C of the 296 locus and allele deletion of the 1960–1977 locus in *KISS1* gene and high litter size in Jining Grey goats [23]. Hou et al. (2011) identified T2643C and 8bp base deletions (2677AGTTCCCC) in the intron 2 of goat *KISS1* gene and the T2643C had significant effects on litter size (*P* < 0.05) [25].

The reproductive traits are complex quantitative traits involving multiple genes, loci and interactions, so it is important to analyze the combined effect of multiple genes or loci on reproductive traits. In the study, association between multiple locus and litter size from the first to the fourth parity was analyzed. Mean litter size of goat tended to increase in later parity. Individuals with SC1 (*AATTAATT*) had higher litter size than those with SC4 (*AATTGACT*) and SC10 (*GGTTAATT*) in the second parity of SN breed. In addition, individuals with SC1 (*AATTAATT*) had higher litter size than those with SC10 (*GGTTAATT*) in average parity of SN breed. The litter size at second kidding is often a valuable index to determine whether a goat is prolific [26]. Therefore, SC1 (*AATTAATT*) can be used in marker-assisted selection to select the individuals with higher litter size. Accumulating evidence further showed that central or peripheral administration of kisspeptin stimulates GnRH-dependent luteinizing hormone (LH) and follicle-stimulating hormone (FSH) secretion in various mammalian species from rodents to humans [27-29], suggesting that kisspeptin plays an essential role in governing reproductive functions throughout species. The biochemical and physiological functions, together with the results obtained in our study, indicate that *KISS1* gene could be as a molecular breeding marker in goats.

## Conclusions

This study explored the genetic polymorphism of *KISS1* gene, and indicated that four SNPs may play an important role in litter size. Their genetic mechanism of reproduction in goat breeds should be further investigated. The female goats with SC1(*AATTAATT*) and BC7(*TTAATT*) had higher litter size than those with other combination genotypes in average parity and could be used for the development of new breeds of prolific goats. Further research on a large number of animals is required to confirm the link with increased prolificacy in goats.

## Methods

### Animals and genomic DNA isolation

Blood samples were obtained from 680 goats belonging to three breeds: Xinong Saanen goat (SN; *n*=257), Guanzhong goat (GZ, *n*=231) and Boer goat (BG; *n*=192). They were reared in Qianyang, Zhouzhi and Liuyou county of Shaanxi province, respectively. All diets were based on alfalfa, corn silage, and a combination of concentrates including corn, soya meal, and bone meal. Health, fertility and production records were maintained by the dairymen and veterinarians. The litter size from the first to fourth parity was obtained from production records. Five milliliters blood per goat were collected aseptically from the jugular vein and kept in a tube containing anticoagulant ACD (citric acid:sodium citrate:dextrose – 10: 27: 38). All samples were delivered back to the laboratory in an ice box. The genomic DNA was extracted from white blood cells using standard phenol-chloroform extraction protocol. All experiments were performed in accordance with the National Institute of Health Guide for the Care and Use of Laboratory Animals.

### SNPs investigation and genotyping

According to caprine *KISS1* gene (GenBank accession no. GU142847), Seven pairs of primers were designed to amplify caprine *KISS1* gene. Their optimal annealing temperatures are showed in Additional file 1: Table S7. Herein we screened them for identifying SNPs of this gene by DNA pooling sequencing assay [30]. Five microliters DNA (100ng/μl) per sample were collected to create a DNA pool for each goat breed. PCR products were sent to Beijing Genomics Institute (Beijing, China) to sequence in both directions. Discovery of SNPs was conducted using Chromas 2.31 and DNAstar 7.0 software.

The SNP in 5'UTR (89-409bp) of *KISS1* gene was genotyped using primer–introduced restriction analysis–polymerase chain reaction (PIRA-PCR) [31]. Other SNPs of *KISS1* gene were genotyped with polymerase chain reaction–restriction fragment length polymorphism (PCR-RFLP). The 25 μL volume contained 50 ng genomic DNA, 12.5 μL 2 × reaction mix (including 500 μM dNTP each; 20 mM Tris–HCl; pH 9; 100 mM KCl; 3 mM MgCl2 ), 0.5 μM of each primer, and 0.5 units of *Taq* DNA polymerase. The cycling protocol was 5 min at 95°C, 35 cycles of denaturing at 94°C for 30 s, annealing at X°C (Additional file 1: Table S7) for 30 s, extending at 72°C for 35 s, with a final extension at 72°C for 10 min. PCR products (5μl) of different primer pairs were mixed with 0.7 μl 10 × Buffer, 2.5 U restriction enzyme (NEB, Ipswich, Britain) and 3.8 μl sterilized ddH_2_O, and then incubated for 1.5 h at 37°C. The restriction enzymes were showed in Additional file 1: Table S1. Digestion products were subjected to 3.5% horizontal agarose gel electrophoresis or 12% polyacrylamide gel electrophoresis (PAGE). The agarose and polyacrylamide gels were stained with ethidium bromide and 0.1% silver nitrate, respectively, and then the genotypes were observed.

The allelic frequencies, heterozygosity (He) and polymorphism information content (PIC) were calculated using Popgene (version 1.31). The linkage disequilibrium was performed by SHEsis software [19]. Association analysis of combinative genotypes of different loci in *KISS1* gene with litter size was done in three goat breeds. SC_n_ ,GC_n_ and BC_n_ represented different combinative genotypes of SN, GZ and BG breeds, respectively. Statistical analysis was performed using univariate analysis in the general linear model procedure of SPSS 16 statistical software. Multiple comparisons of the means were performed using the least significant difference method or Dunnett’s T3. The model applied was: *Y*_ikm_ = *μ* + *G*_i_ + *S*_k_*+ E*_ikm_, where *Y*_ikm_ is the trait measured on each of the ikm^th^ animal, *μ* is the overall population mean, *G*_i_ is the fixed effect associated with i^th^ genotype or combinative genotype, *S*_k_ is the fixed effect associated with the k^th^ sire, and *E*_ikm_ is the random error. Effects associated with farm, birth year and season of birth are not matched in the linear model, as the preliminary statistical analyses indicated that these effects did not have a significant influence on variability of traits in the analyzed populations.

## Competing interests

The authors declare that they have no competing interests.

## Authors’ contributions

XA, JH and TM conducted all experiments and wrote the manuscript. FF, YY and HZ carried out the computational analysis. PH and YS contributed their ideas towards writing. BCao and JW conducted the research design. All authors read and approved the final manuscript.

## Supplementary Material

Additional file 1: Table S1 Identified SNPs within the *KISS1* gene and positions in reference sequence. **Table S2.** Genotypic distribution and allelic frequencies of four SNP loci in *KISS1* gene. **Table S3.** Linkage disequilibrium (*r*^*2*^) between four SNPs in *KISS1* gene. **Table S4.** Least square means and standard errors of the litter size of SN breed for four locus genotypes in *KISS1* gene. **Table S5.** Least square means and standard errors of the litter size of GZ breed for four locus genotypes in *KISS1* gene. **Table S6.** Least square means and standard errors of the litter size of BG breed for *g.2489T>C*, *g.2510G>A* and *g.2540C>T* locus genotypes. **Table S7.** Primer sequences for goat *KISS1* gene applied for screening polymorphisms and genotyping. Click here for file
