# Supplementary material for: Association analysis between variants in KISS1 gene and litter size in goats
Source: BMC Genet. 2013 Aug 2;14:63. doi: 10.1186/1471-2156-14-63 (PMC3734198; doi:10.1186/1471-2156-14-63)
Supplement: Additional file 1: Table S1 — Identified SNPs within the KISS1 gene and positions in reference sequence. Table S2. Genotypic distribution and allelic frequencies of four SNP loci in KISS1 gene. Table S3. Linkage disequilibrium (r2) between four SNPs in KISS1 gene. Table S4. Least square means and standard errors of the litter size of SN breed for four locus genotypes in KISS1 gene. Table S5. Least square means and standard errors of the litter size of GZ breed for four locus genotypes in KISS1 gene. Table S6. Least square means and standard errors of the litter size of BG breed for g.2489T>C, g.2510G>A and g.2540C>T locus genotypes. Table S7. Primer sequences for goat KISS1 gene applied for screening polymorphisms and genotyping. [file 1471-2156-14-63-S1.doc]

**On-line supplementary data**

**Table S1** Identified SNPs within the *KISS1* gene and positions in reference sequence

| Primer | SNP | Gene  region | Variation relative to reference sequence | Restriction enzyme | Position on reference sequence (GU142847) | SNP accession number  (GenBank accession no.) |
| --- | --- | --- | --- | --- | --- | --- |
| KISS1-F2 | **g.384G>A** | 5'UTR (89-409) | G>A | *Mwo*I | 384 | JQ796693 |
| KISS1-R2B |  |  |  |  |  |  |
| KISS1-F4  KISS1-R4 | g.1147T>C | Intron 1  (987-1566) | T>C | - | 1147 | JQ796693 |
|  | g.1417G>A |  | G>A |  | 1417 |  |
|  | g.1428_1429delG |  | DelG |  | 1429 |  |
| KISS1-F5 | g.2124T>A | Intron 1  (2000-2376) | T>A | - | 2124 | JQ806381 |
| KISS1-R5 | g.2270C>T |  | C>T |  | 2270 |  |
| KISS1-F6  KISS1-R6 | **g.2489T>C** | Intron 1  (2357-2598) | T>C | *SfaN*I | 2489 | JQ806381 |
|  | **g.2510G>A** |  | G>A | *Alu*I | 2510 | JQ806382 |
|  | **g.2540C>T** |  | C>T | *Sac*I | 2540 |  |
| KISS1-F7 | g.3864_3865delCA | 3'UTR  (3678-4099) | DelCA | - | 3865 | JQ809704 |
| KISS1-R7 | g.3885_3886insACCCC |  | InsACCCC |  | 3886 |  |

*Note*: SNPs marked in bold were included in the association analysis. Genomic positions of the SNPs refer to caprine *KISS1* gene sequence (GenBank accession no. GU142847)

**Table S2** Genotypic distribution and allelic frequencies of four SNP loci in *KISS1* gene

| Locus | | | Breed | | |
| --- | --- | --- | --- | --- | --- |
|  |  |  | SN | GZ | BG |
| *g.384G>A* | Genotype | *GG* | 69 | 67 | 192 |
|  |  | *GA* | 144 | 130 | 0 |
|  |  | *AA* | 44 | 34 | 0 |
|  | Allele | *G* | 0.55 | 0.57 | 1 |
|  |  | *A* | 0.45 | 0.43 | 0 |
|  | He | | 0.56 | 0.56 | - |
|  | PIC | | 0.37 | 0.37 | - |
|  | Equilibrium  *χ*^2^ test | | *P*=0.04 | *P*=0.02 | - |
| *g.2489T>C* | Genotype | *TT* | 165 | 126 | 127 |
|  |  | *TC* | 92 | 105 | 65 |
|  | Allele | *T* | 0.82 | 0.77 | 0.83 |
|  |  | *C* | 0.18 | 0.23 | 0.17 |
|  | He | | 0.36 | 0.45 | 0.34 |
|  | PIC | | 0.25 | 0.29 | 0.24 |
|  | Equilibrium  *χ*^2^ test | | *P*<0.01 | *P*<0.01 | *P*<0.01 |
| *g.2510G>A* | Genotype | *GG* | 32 | 58 | 32 |
|  |  | *GA* | 130 | 118 | 89 |
|  |  | *AA* | 95 | 55 | 71 |
|  | Allele | *G* | 0.38 | 0.51 | 0.40 |
|  |  | *A* | 0.62 | 0.49 | 0.60 |
|  | He |  | 0.51 | 0.51 | 0.46 |
|  | PIC |  | 0.36 | 0.38 | 0.36 |
|  | Equilibrium  *χ*^2^ test |  | *P*=0.22 | *P*=0.74 | *P*=0.65 |
| *g.2540C>T* | Genotype | *CC* | 42 | 47 | 40 |
|  |  | *CT* | 121 | 108 | 89 |
|  |  | *TT* | 94 | 76 | 63 |
|  | Allele | *C* | 0.40 | 0.44 | 0.44 |
|  |  | *T* | 0.60 | 0.56 | 0.56 |
|  | He | | 0.47 | 0.47 | 0.46 |
|  | PIC | | 0.36 | 0.37 | 0.37 |
|  | Equilibrium  *χ*^2^ test | | *P*=0.77 | *P*=0.45 | *P*=0.41 |

**Table S3** Linkage disequilibrium (*r^2^*) between four SNPs in *KISS1* gene

| Locus | Breed | | |
| --- | --- | --- | --- |
|  | SN | GZ | BG |
| *g.384G>A* and *g.2489T>C* | 0.05 | 0.01 | - |
| *g.384G>A* and *g.2510G>A* | 0.03 | 0.01 | - |
| *g.384G>A* and *g.2540C>T* | 0.02 | 0.01 | - |
| *g.2489T>C* and *g.2510G>A* | 0.29 | 0.19 | 0.15 |
| *g.2489T>C* and *g.2540C>T* | 0.22 | 0.10 | 0.11 |
| *g.2510G>A* and *g.2540C>T* | 0.68^a^ | 0.42^a^ | 0.41^a^ |

*Note:* LD = linkage disequilibrium. ^a^ represents strong linkage disequilibrium (*r*^2^ > 0.33).

**Table S4** Least square means and standard errors of the litter size of SN breed for four locus genotypes in *KISS1* gene.

| Breed | Combinative  genotype | Number | 1^st^ parity  litter size | 2^nd^ parity  litter size | 3^rd^ parity  litter size | 4^th^parity  litter size | Average  litter size |
| --- | --- | --- | --- | --- | --- | --- | --- |
| SN | SC1(*AATTAATT*) | 18 | 1.49±0.14^a^ | 2.18±0.12^bc^ | 2.17±0.14 | 2.52±0.16^b^ | 2.04±0.08^bc^ |
|  | SC2(*AATTGGCC*) | 9 | 1.51±0.19 | 2.05±0.17 | 2.48±0.21^b^ | 1.90±0.23^a^ | 1.97±0.11 |
|  | SC3(*AATCGACT*) | 5 | 2.15±0.29^b^ | 2.42±0.23^b^ | 2.15±0.27 | 2.27±0.33 | 2.30±0.15^b^ |
|  | SC4(*AATTGACT*) | 8 | 1.61±0.20 | 1.65±0.18^ae^ | 2.00±0.21 | 1.95±0.23^a^ | 1.84±0.12^ac^ |
|  | SC5(*GATCGGCC*) | 8 | 1.44±0.20^a^ | 1.83±0.18^acd^ | 1.71±0.21^a^ | 2.36±0.24 | 1.78±0.12^ac^ |
|  | SC6(*GATTAACT*) | 10 | 1.81±0.18 | 1.90±0.17 | 2.39±0.19^bc^ | 2.26±0.22 | 2.03±0.11 |
|  | SC7(*GATCGACT*) | 36 | 1.39±0.10^a^ | 1.99±0.09^bce^ | 2.08±0.10 | 2.20±0.12 | 1.94±0.06^ac^ |
|  | SC8(*GATTGACT*) | 29 | 1.35±0.11^a^ | 2.05±0.10^bce^ | 1.85±0.11^a^ | 2.18±0.13 | 1.87±0.06^ac^ |
|  | SC9(*GATTAATT*) | 32 | 1.40±0.10^a^ | 2.00±0.09^bce^ | 2.07±0.11 | 2.18±0.13 | 1.92±0.06^ac^ |
|  | SC10(*GGTTAATT*) | 28 | 1.52±0.11^a^ | 1.73±0.10^a^ | 1.93±0.11^a^ | 2.28±0.13 | 1.84±0.06^a^ |
|  | SC11(*GGTCGACT*) | 26 | 1.63±0.11 | 1.96±0.10 | 1.98±0.12^ac^ | 2.23±0.13 | 1.92±0.07^ac^ |
|  | SC12(*GGTTGGCC*) | 5 | 1.38±0.26^a^ | 1.78±0.23 | 2.13±0.27 | 2.14±0.30 | 1.95±0.15 |
|  | SC13(*GATTGGCC*) | 5 | 1.14±0.26^a^ | 1.88±0.23 | 1.96±0.27 | 2.21±0.30 | 1.85±0.15^ac^ |
|  | SC14(*GATTGATT*) | 8 | 1.32±0.20^a^ | 2.31±0.18^bd^ | 1.72±0.21^a^ | 2.06±0.24 | 1.81±0.12 |

*Note*: Values with different superscripts in the same column differ significantly at *P* < 0.05.

**Table S5** Least square means and standard errors of the litter size of GZ breed for four locus genotypes in *KISS1* gene.

| Breed | Combinative  genotype | Number | 1^st^ parity  litter size | 2^nd^ parity  litter size | 3^rd^ parity  litter size | 4^th^parity  litter size | Average  litter size |
| --- | --- | --- | --- | --- | --- | --- | --- |
| GZ | GC1(*AATTAACT*) | 6 | 1.43±0.23 | 1.27±0.21 | 1.93±0.24 | 1.87±0.23 | 1.54±0.11 |
|  | GC2(*GATCGGCC*) | 9 | 1.78±0.18^b^ | 1.79±0.18 | 1.51±0.17^ac^ | 1.60±0.18 | 1.69±0.09 |
|  | GC3(*GATCGACT*) | 36 | 1.41±0.09 | 1.64±0.08 | 1.86±0.09^ab^ | 1.92±0.09 | 1.72±0.04 |
|  | GC4(*GATTGACT*) | 28 | 1.41±0.10 | 1.74±0.10 | 1.81±0.10 | 1.99±0.10 | 1.73±0.05 |
|  | GC5(*GATTGGCT*) | 6 | 1.66±0.23^bc^ | 1.34±0.21 | 2.01±0.21^abd^ | 2.03±0.22 | 1.75±0.11 |
|  | GC6(*GATTAATT*) | 22 | 1.51±0.11^bc^ | 1.53±0.11 | 1.55±0.11^cd^ | 1.95±0.12 | 1.66±0.06 |
|  | GC7(*GATCGATT*) | 9 | 1.45±0.17 | 1.76±0.17 | 2.09±0.17^b^ | 1.82±0.18 | 1.78±0.09 |
|  | GC8(*GATTGATT*) | 5 | 1.47±0.24 | 1.51±0.23 | 1.99±0.23 | 1.97±0.24 | 1.75±0.12 |
|  | GC9(*GATTGGTT*) | 6 | 1.33±0.21 | 1.65±0.21 | 1.78±0.21 | 1.69±0.22 | 1.63±0.11 |
|  | GC10(*GGTCGGCC*) | 8 | 1.50±0.19 | 1.68±0.18 | 1.46±0.18^c^ | 1.58±0.19 | 1.59±0.09 |
|  | GC11(*GGTTGGCC*) | 8 | 1.29±0.18 | 1.77±0.18 | 1.89±0.18 | 1.71±0.19 | 1.66±0.09 |
|  | GC12(*GGTCGACT*) | 17 | 1.10±0.13^a^ | 1.54±0.12 | 1.93±0.13^ab^ | 2.01±0.13 | 1.63±0.06 |
|  | GC13(*GGTTAATT*) | 16 | 1.56±0.13^bc^ | 1.63±0.13 | 1.66±0.13^ac^ | 1.90±0.13 | 1.69±0.06 |
|  | GC14(*GGTCGATT*) | 7 | 1.18±0.20^ac^ | 1.71±0.19 | 1.44±0.19^c^ | 1.78±0.21 | 1.54±0.10 |

*Note*: Values with different superscripts in the same column differ significantly at *P* < 0.05.

**Table S6** Least square means and standard errors of the litter size of BG breed for *g.2489T>C*, *g.2510G>A* and *g.2540C>T* locus genotypes.

| Breeds | Combinative  genotype | Number | 1^st^ parity  litter size | 2^nd^ parity  litter size | 3^rd^ parity  litter size | 4^th^parity  litter size | Average  litter size |
| --- | --- | --- | --- | --- | --- | --- | --- |
| BG | BC1(*TTAACC*) | 10 | 1.31±0.15 | 1.87±0.17 | 2.01±0.17 | 2.26±0.15 | 1.80±0.09 |
|  | BC2(*TCGGCC*) | 13 | 1.48±0.13 | 1.86±0.15 | 1.70±0.14 | 1.87±0.13^a^ | 1.71±0.08 |
|  | BC3(*TTGGCC*) | 13 | 1.22±0.14 | 1.51±0.16 | 1.94±0.14 | 1.98±0.13 | 1.65±0.08 |
|  | BC4(*TTAACT*) | 11 | 1.08±0.15 | 1.83±0.16 | 1.88±0.16 | 1.93±0.15^a^ | 1.66±0.08 |
|  | BC5(*TCGACT*) | 41 | 1.32±0.08 | 1.74±0.08 | 1.82±0.08 | 2.01±0.08^a^ | 1.72±0.04 |
|  | BC6(*TTGACT*) | 32 | 1.20±0.09 | 1.75±0.10 | 1.65±0.10^a^ | 1.94±0.09^a^ | 1.59±0.05^a^ |
|  | BC7(*TTAATT*) | 42 | 1.46±0.08 | 1.76±0.09 | 2.01±0.09^b^ | 2.26±0.08^b^ | 1.86±0.04^b^ |
|  | BC8(*TTGATT*) | 11 | 1.17±0.15 | 1.70±0.16 | 1.83±0.15 | 2.14±0.14 | 1.68±0.08 |

*Note*: Values with different superscripts in the same column differ significantly at *P* < 0.05.

**Table S7 Primer sequences for goat *KISS1* gene applied for screening polymorphisms and genotyping**

| Primer | Sequence (5'→3') | Gene region | Amplicon  (bp) | ^a^Position in reference  sequence | Tm (℃) |
| --- | --- | --- | --- | --- | --- |
| KISS1-F1 | TTGCTGGACAGTCTCAAAG | 5'UTR | 209 | 8-216 | 50 |
| KISS1-R1 | TGTCTCCACATTCTGTTCTC |  |  |  |  |
| KISS1-F2 | TGGAGGATGAGAAGAGAAGG | 5'UTR | 347 | 89-435 |  |
| KISS1-R2A | CTCGGCTTTGCATGAATCAC |  |  |  | 61.5 |
| ^b^KISS1-R2B | TAGTACTCCACTCTCGCAGC |  | 321 |  | 56 |
| KISS1-F3 | TGCAAAGCCGAGTGTGCAGG | 5'UTR | 594 | 424-1017 | 65 |
| KISS1-R3 | TGAAGGCGGTGGCACAAAGG | -Exon 1 |  |  |  |
| KISS1-F4 | CTGATGCTCTTCCTTTGTGC | Exon 1 | 579 | 987-1566 | 56 |
| KISS1-R4 | GTTTCTGCAGCCTTGGATTG | Intron 1 |  |  |  |
| KISS1-F5 | CCCGCTGTAACTAGAGAAAG | Intron 1 | 377 | 2000-2376 | 51 |
| KISS1-R5 | CATCCAGGGTGAGTGATACT |  |  |  |  |
| KISS1-F6 | AGTATCACTCACCCTGGATG | Intron 1 | 242 | 2357-2598 | 51 |
| KISS1-R6 | CACTTCACATACAAGGCTCAT |  |  |  |  |
| KISS1-F7 | ATGCTGCCGAATCCTCTGCGT | 3'UTR | 422 | 3678-4099 | 64 |
| KISS1-R7 | TTAAGCCAGCTGCCTTCCCTC |  |  |  |  |

^a^ Location of primers referred to the sequence of goat *KISS1* gene (GenBank accession no. GU142847). ^b^KISS1-R2B and KISS1-F2 were used in primer–introduced restriction analysis–polymerase chain reaction (PIRA-PCR). KISS1-R2B primer was deliberately introduced into a point mutation (G) in order to create a *Mwo*I restriction site.
